# Beef Tea and Meat Preparations

**Published:** 1902-02-01

**Authors:** Marcus R. P. Dorman


### The Institutional Workshop.

### BEEF TEA AND MEAT PREPARATIONS.

By Marcus R. P. Dorman, M.D.

### II.—BEEF TEA AND MEAT PREPARATIONS COMPARED.

The weight of the total solids in beef tea differs very considerably according to the method which is adopted in brewing. Dr. Bedson made his beef tea in what he calls "the ordinary way," viz.; he allowed very nearly a pound of beef to soak in a pint of cold water for some hours, and then gradually warmed it to near boiling point, and obtained 229·29 grains of total solids. Dr. Rideal took one pound of American beef to the pint of water, infused it for an hour in the cold, and then simmered it in closed jars in the oven for six hours, and obtained 318·5 grains of total solids.[1] It is, therefore, very difficult to say what is the average strength of beef tea as usually prepared, but it would probably be nearer that of Dr. Bedson's sample than that of Dr. Rideal's. For purposes of these calculations then we shall assume that 250 grains of total solids to the pint is the strength of ordinary beef tea, as given in hospitals and to private patients. This total includes, of course, not only the extractives proper, but the salts and the small amount of albumen. When we examine the commercial meat preparations we are on much firmer ground, for the amount of water varies but little in different samples, and it is therefore easy to discover the proportion of total solids in any given weight used. This represents the total of the extractives, salts, fat, and albumens. But since the various preparations vary considerably in the amount of water they contain, this must be discovered and calculated before we can know the weight to be used in order to make a solution of any standard strength. The following table[2] shows the difference in three well-known preparations:—

| Name. | Total dry solids. |
|---|---|
| Armour's ... ... ... ... | 78·1 |
| Bovril ... ... ... ... ... | 62 8 |
| Lemco ... ... ... ... ... | 32 2 |

The following formula will give us in the case of a pure extract the weight necessary. Let X equal the weight of extract in grains which will form a solution having 250 grains of total solids to the pint of water and Y the percentage of solids in the extract then $X = \frac{100}{Y} \times 250$. For Lemco and Armour's Extract the sum works out at rather more than ⅙ oz. This formula cannot obviously be applied to Bovril or other preparation in which part of the total solids is added albumin and fibrine, but about the same percentage of flesh bases is found in Bovril as in Armour's extract.

It is impossible to compare the complete analyses unless they are performed by the same chemist, for each one expresses his results in different terms; but we must always be cautious of regarding the amount of nitrogen as an indication of the useful extracts present, since this element may be partly furnished by urea which contains nearly 47 per cent. of its weight of nitrogen, whereas creatine contains only about 32 per cent. Mr. A. McGill, B.A., who carried out the analyses for the Ottawa Inland Revenue Department above referred to, thus writes: "Unfortunately I have been unable to discover any practical method of working by which a sharp line can be drawn between the nitrogen present as urea and that present as creatine, creatinin and xanthin. This is one of many points which have been suggested in this examination which demand further work." It seems therefore that the total solids is the only safe denominator we can use, but since the chief characteristics of all extracts is that they chiefly consist of flesh bases, they are, therefore, allied in composition to beef tea, and differ from nourishing preparations. They are therefore chiefly stimulants, and the manner in which they act is briefly as follows: the nitrogen in lean beef is combined with carbon, hydrogen, oxygen, and sulphur to form complex compounds called proteids, substances which differ greatly among themselves, but which may be characterised as more or less closely allied to white of egg. These are converted into peptones by the action of the gastric juices, but this process of peptonisation can also be effected outside the stomach by means of pepsin, usually obtained from the stomachs of pigs. The proteid matter then undergoes many changes in the tissues, and is ultimately eliminated by the kidneys, lungs, and skin, chiefly in the form of urea, water, and carbonic acid. This process, however, is a very complex one, many compounds being formed on the way, which are known as flesh bases, of which creatine may be taken as a type, since it exists in largest amount. These chemical changes are accompanied by the liberation of a great deal of energy, the whole of the proteid tissues of the body (muscle, nerves, etc.) so to speak continually wearing out. The urea and other waste products are then at once removed by the blood-stream, and nourishing material is supplied in their place by the same agency. It is therefore obvious that, although the flesh bases as found in beef-tea and extracts are not in themselves food, nevertheless they still have several changes to undergo before they are finally eliminated as urea, during which period much vital force is liberated. For this reason beef-tea and meat-extracts are suitable for all cases requiring a temporary manifestation of extra vital energy, but they must never be regarded as a substitute for nourishing food. The rule of thumb diet of three pints of milk and one of beef-tea or extract per diem in enteric fever is thus perfectly rational, the former being food and the latter stimulant. In acute diseases, and after collapse from hæmorrhage or shock following accidents or operations, it is often desirable to administer strong doses of stimulants, and then the utility of the meat-extract is far greater than that of beef-tea, since practically any strength can be at once made and given. There are many other details of importance, such as the presence or absence of chemical preservatives and the amount of added sodium chloride, but, speaking generally, we can say that the freer the extract from preservatives the easier it is to digest.

[1] Hospital Expenditure: The Commissariat, pp. 110, 111. The fat was omitted in both cases.

[2] The analyses of Armour's and Bovril are taken from Bulletin 63, Laboratory of the Inland Revenue Department, Ottawa, Canada, 1899. The analysis of Lemco is from *Science Siftings*, February 3, 1900.

